# Interplay between cross sectional analysis of risk factors associated with *Toxoplasma gondii* infection in pregnant women and their domestic cats

**DOI:** 10.3389/fvets.2023.1147614

**Published:** 2023-03-24

**Authors:** Eman A. A. Hassanen, Dennis N. Makau, Mohamed Afifi, Omar A. Al-Jabr, Mohammed Abdulrahman Alshahrani, Ahmed Saif, Reham G. A. Anter, Wafaa M. El-Neshwy, Doaa Ibrahim, Rasha M. M. Abou Elez

**Affiliations:** ^1^Department of Parasitology, Faculty of Veterinary Medicine, Zagazig University, Zagazig, Egypt; ^2^Department of Veterinary Population Medicine, University of Minnesota, St. Paul, MN, United States; ^3^Department of Animal Wealth Development, Biostatistics Section, Faculty of Veterinary Medicine, Zagazig University, Zagazig, Egypt; ^4^Department of Health Management, Atlantic Veterinary College, University of Prince Edward Island, Charlottetown, PEI, Canada; ^5^Department of Microbiology, College of Veterinary Medicine, King Faisal University, Al-Ahsa, Saudi Arabia; ^6^Department of Clinical Laboratory Sciences, Faculty of Applied Medical Sciences, Najran University, Najran, Saudi Arabia; ^7^Department of Clinical Laboratory Sciences, Faculty of Applied Medical Sciences, Najran University, Najran, Saudi Arabia; ^8^Department of Animal Medicine, Division of Infectious Diseases, Faculty of Veterinary Medicine, Zagazig University, Zagazig, Egypt; ^9^Department of Nutrition and Clinical Nutrition, Faculty of Veterinary Medicine, Zagazig University, Zagazig, Egypt; ^10^Department of Zoonoses, Faculty of Veterinary Medicine, Zagazig University, Zagazig, Egypt

**Keywords:** toxoplasma, PCR, cat, human, risk factor

## Abstract

Toxoplasmosis is a global zoonotic disease caused by *Toxoplasma gondii* (*T. gondii*). The primary aim of this study was to identify hygienic and cat management practices that could affect the occurrence of *T. gondii* in cats and their owners in Sharqia Governorate, Egypt. *T. gondii* infection was evaluated in 80 pregnant women and 29 domestic cats using Real-time PCR. A questionnaire was administered to obtain information regarding the risk factors associated with *T. gondii* infection. Blood samples were collected from enrolled pregnant women, and fecal samples were collected from their pet cats. Firth logistic regression model complemented with random forest (RF) analysis was used to evaluate the association of different hygiene and cat management practices with *T. gondii* infection in pregnant women. *T. gondii* infection was identified in 27.5% of pregnant women and 17% of domestic cats. Drinking raw milk and contacting stray and pet cats were significantly associated with higher odds of *T. gondii* infection. The proportion of *T. gondii*-positive women who ate raw meat (60.0%) was higher than those ate heat-cooked meat (25.3%). Moreover, women who did not wash their hands after contact with cats were 6 times (OR 6.12; CI: 3.03–9.21) more likely to experience *T. gondii* infection than those washed their hands after cat contact. The RF analysis showed that washing hands constitute a serious yet preventable public health concern that requires targeted, community-specific efforts. Cat owners, particularly pregnant women, need to be aware about the risk of *T. gondii* infection, while handling cat and pet's owner should be advised to take necessary hygienic measures to prevent its infection.

## 1. Introduction

*Toxoplasma gondii* is an obligatory intracellular protozoan parasite that causes worldwide toxoplasmosis in all warm-blooded animals and humans. Previous meta-analyses showed pooled prevalence rates of 1.1 and 33.8% for acute and latent *Toxoplasma* infection in pregnant women, respectively (1, 2). The prevalence of human toxoplasmosis substantially varies from one region to other (3, 4). Previously, reported risk factors included the type and the cooking method of food, adequate treatment for drinking water, contact with cats being immunocompromised (5–7). In Egypt, similar risk factors were previously reported (8–14). The life cycle of *T. gondii* includes final hosts (i.e., the Felidae family, mainly cats), where the sexual cycle of replication of *T. gondii* oocytes occurs and intermediate hosts (i.e., non-feline including dogs and humans). The intermediate hosts primarily are infected by consuming undercooked meat or food contaminated with oocytes excreted by cats (15). Clinically, toxoplasmosis is most often in apparent in humans; however, clinical symptoms sometimes become critical particularly when congenital or postnatal infection occurs. The congenital infection mainly occurs in the first trimester, when pregnant women are more susceptible to *T. gondii* compared to the rest of the pregnancy stages (16, 17). Congenital toxoplasmosis might also affect the central nervous system resulting in neurological complications and ocular lesions (18).

In most cases, toxoplasmosis is a latent infection; however, non-specific clinical signs could also appear. Therefore, diagnosing *T. gondii* using clinical signs might be infeasible. Alternatively, serological tests such as the direct agglutination test, the latex agglutination test, and ELISA are commonly used to detect humoral antibodies (19). Moreover, molecular identification using PCR has been showing higher sensitivity and specificity than serological assays (20–23). Varying levels of toxoplasmosis seroprevalence in Egyptian pregnant women, ranging from 3.8 to 67.5%, have been reported across different governorates (9, 10, 13, 21, 24–27), with a pooled rate of 45.9% (2). Sharqia Governorate is Egypt's third most populous governorate (28), and it has been experiencing an increasing trend of keeping domestic cats and a growing number of stray cats. Health and hygiene practices have also been challenging, especially in the rural areas of the Governorate. A previous study focused on the molecular identification of *T. gondii* DNA in the milk samples from goats, sheep and cows in the governate (27). However, no study has addressed the molecular positivity of *T. gondii* in both pregnant women and cats in this area. The primary aim of this study was to identify hygienic and cat management practices affecting the occurrence of *T. gondii* in cats and pregnant women in Sharqia Governorate, Egypt.

## 2. Materials and methods

### 2.1. Patients and study area

Data on the hygiene and cat management practices associated with *T. gondii* infection were collected using a structured questionnaire adapted from previous studies (9, 29). The main domains of the questionnaire included socio-demographic characteristics, hygienic practices, and cat management factors. The questionnaire was initially drafted in Arabic; however, a full English-translated version is available in in a Supplementary Table S1. The questionnaire was distributed among 80 pregnant women admitted to Al-Ahrar hospital and Minya-Elqamh hospital in the Sharqia governorate, Egypt. Written consent was obtained from all participating women, and they were also asked about their willingness to enroll their pet cats in the study.

### 2.2. Collection of human blood and cat fecal samples

Peripheral blood samples were collected from all enrolled women and stored at −20°C for DNA extraction. Blood samples were collected on EDTA as anticoagulant (1 mg/mL). The participants who agreed to enroll their pet cats into the study were advised to collect fresh feces into an air tight plastic bag and store them at 4°C for transportation to the Laboratory of Parasitology, Faculty of Veterinary Medicine, Zagazig University. This study followed the guidance of the Research, Publication, and Ethics Committee of the Faculty of Veterinary Medicine, Zagazig University, Egypt (ethical approval number: ZU-IACUC/2/F/205/2022).

### 2.3. Fecal samples processing

The oocytes of *T. gondii* were recovered using the floataion centrifugation technique. Three grams of fecal samples were mixed with distilled water in a centrifuge tube and spun at 3,000 rpm for 5 min. The clear supernatant was discarded, and saturated NaCl solution was added until ¾ of the tube's height; then, the tube was centrifuged again at 3,000 rpm for 5 min. Subsequently, we filled the tubes with saturated NaCl and let them sit for 20 min. We carefully touched a glass slide on the water surface, then covered it with a cover slide and examined the presence of thick-walled unstained oocysts under the microscope (400 ×) (30). The examined positive and negative fecal samples were stored at −20°C for confirmation by molecular identification.

### 2.4. Real-time PCR of *T. gondii*

Quantitative molecular identification was used to identify the B1 gene. The QIAamp DNA Mini Kit (Qiagen, Germany, GmbH) was used to extract DNA according to the producer's instructions, followed by ten cycles of freezing (in liquid nitrogen) and thawing (in a water bath at 60°C) to disrupt the oocytes. Briefly, 200 μL from each fecal suspension and blood sample were incubated with 10 μL of proteinase K and 200 μL of lysis buffer at 56°C for 10 min. After incubation, 200 μL of absolute ethanol was added to the lysate. The sample was then washed and centrifuged following the manufacturer's recommendations. The nucleic acid was eluted with 100 μL of elution buffer provided in the kit. The oligonucleotide primer supplied from Metabion, Germany was used. The primer sequences used in this study and PCR cycle conditions carried out in Strata gene MX3005P RT PCR machine (31) are available in a Supplementary Table S2. The extracted DNA samples were examined by Real-time PCR using primers in a 25 μL reaction containing 12.5 μL of 2 × QuantiTect SYBR Green PCR Master Mix (Qiagen, Gmbh), 1 μL of each primer of 20 pmol concentration, 4.5 μL of water, and 6 μL of DNA template. A positive control *T. gondii* strain was kindly obtained from the biotechnology department, Animal Health Research Institute, Dokki, Giza, and a reaction mixture with no added DNA was run in the PCR reaction as positive and negative controls, respectively.

### 2.5. Data analysis

*Toxoplasma gondii* positivity among pregnant women was modeled as a categorical outcome encoded as zero for negative individuals and one for cases. Two complementary analyses were performed on the data. We used the penalized maximum likelihood estimation in a Firth logistic regression framework and random forest supervised machine learning algorithms. Due to the small sample size and few positive cases, estimates from conventional logistic regressions had wide confidence intervals (CI). We ran univariable regression models fitting each risk factor as an independent variable and variables associated with the outcome at a *p*-value ≤ 0.1 were subsequently included in the multivariable model. Wald's test was used to evaluate the overall significance of categorical variables with more than 2 levels. Results were presented as odds ratios (OR) and 95% CI. We found no evidence of confounding or significant interaction among the tested variables.

A random forest (RF) classification analysis was also applied to complement the statistical model so that inferences would not be solely based on the *p*-values (32). The RF analysis allows for a multi-way comparison of all independent variables; it uses different metrics to weigh the relative importance of each variable in explaining variability in the outcome (33, 34).

In the RF model, we split the data such that 80% was used for training and parametrising the model, and the remaining 20% was used to make predictions and test model performance. After model tuning using hyperparameters, we ran 500 iterations and evaluated model performance and tuning using accuracy (overall proportion of observations correctly classified as *T. gondii* positive and negative), sensitivity (proportion of *T. gondii* positive cases correctly classified), and specificity (proportion of *T. gondii* negative correctly classified). Additionally, we generated variable importance plots, which ranked specific model covariates based on their influence on model accuracy and the Gini index, which represents when the data was split based on a given predictor, and how homogenous were the *T. gondii* positive and negative groups.

## 3. Results

The study recruited 80 pregnant women from two hospitals, with 22 women (27.5%) testing positive for *T. gondii*. The age of women ranged from 18 to 50 years, and 48 (60%) of the 80 were residing in rural areas. Complete demographic data are presented in Table 1.

**Table 1:** Demographic characteristics of the enrolled women and univariable firth logistic regression model.

	**–Ve, *N* (%)^a^**	**+Ve, *N* (%)**	**OR (95% CI)**
**Age** ^b^			
18–24	18 (72.0%)	7 (28.0%)	–
25–35	29 (72.5%)	11 (27.5%)	0.96 (0.32–2.85)
>35	11 (73.3%)	4 (26.7%)	0.97 (0.24–3.84)
**Residence**			
Rural	31 (64.6%)	17 (35.4%)	–
Urban	27 (84.4%)	5 (15.6%)	0.36 (0.12–1.07)
**Occupation** ^b^			
Student	10 (90.9%)	1 (9.1%)	–
Housewife	29 (82.9%)	6 (17.1%)	1.54 (0.23–10.42)
Employee	7 (58.3%)	5 (41.7%)	5.13 (0.67–39.25)
Farmer	12 (54.6%)	10 (45.5%)	5.88 (0.88–39.21)
**Education** ^b^			
Unable to read and write	6 (27.3%)	16 (72.7%)	–
Low education school	10 (71.4%)	4 (28.6%)	0.17 (0.04–0.71)
High education school	42 (95.5%)	2 (4.6%)	0.02 (0.00–0.11)
**Pregnancy trimester** ^b^			
1st	38 (69.1%)	17 (30.9%)	–
2nd	16 (80.0%)	4 (20.0%)	0.60 (0.18–1.96)
3rd	4 (80.0%)	1 (20.0%)	0.73 (0.11–5.06)
**Blood transfusion history**			
Yes	3 (50.0%)	3 (50.0%)	–
No	55 (74.3%)	19 (25.7%)	0.35 (0.07–1.69)
**Abortion history** ^c^			
Yes	29 (64.4%)	16 (35.6%)	–
No	29 (82.9%)	6 (17.1%)	–

^a^Row percentages.

^b^Overall p-values of Wald tests were 0.997, 0.07, P < 0.001, 0.686.

^c^Abortion history was not considered as a risk factor for T. gondii infection.

OR, odds ratio; 95% CI, confidence interval.

From the univariable analysis, factors significantly associated with *T. gondii* infection included education, where the odds of being *T. gondii* positive decreased by 83 and 98% for low-educated women (i.e., at a lower secondary qualification, at or below International Standard Classification of Education) and high-educated women (i.e., with a Bachelor program, Master degree program, Doctoral degree program at research university), respectively, compared to illiterate women. Employees and farmers showed higher odds (OR: 5.13; 95% CI: 0.67–39.25, and OR: 5.88; 95% CI: 0.88–39.21, respectively) of *T. gondii* infections compared to students; however, there was no sufficient power to identify significant differences. There was also no evidence of significance that women with a history of blood transfusion were more likely to encounter *T. gondii* infection.

Drinking raw milk was significantly associated with higher odds of *T. gondii* infection, such that women drinking raw milk were 5.5 times more likely to experience *T. gondii* infection than women who used pasteurized or processed milk. Also, getting in contact with stray and pet cats was associated with higher odds of *T. gondii* infection (OR 47.4; CI: 6.60–342.40). The proportion of *T. gondii*-positive women who eat raw meat (60.0%) was higher than those who cooked (25.3%). Moreover, women who did not wash their hands after contact with cats were 6 times (OR 6.12; CI: 3.03–9.21) more likely to experience *T. gondii* infection than those who washed their hands after cat contact (Table 2).

**Table 2:** A summary of hygienic practices and contact with cats, univariable firth logistic regression model.

	**–Ve *N* (%)^a^**	**+Ve *N* (%)**	**OR (95%CI)**
**Eating undercooked meat**			
No	56 (74.7%)	19 (25.3%)	–
Yes	2 (40.0%)	3 (60.0%)	4.06 (0.74–22.26)
**Drinking raw milk**			
No	56 (75.7%)	18 (24.3%)	–
Yes	2 (33.3%)	4 (66.7%)	5.5 (1.07–28.13)
**Contact with cats** ^b^			
No contact	30 (96.8%)	1 (3.2%)	–
Pet cat	12 (80.0%)	3 (20.0%)	5.7 (0.80–43.04)
Stray cat	12 (60.0%)	8 (40.0%)	13.8 (2.20–88.50)
Pet and stray cats	4 (28.6%)	10 (71.4%)	47.4 (6.60–342.40)
**Washing hands after cat contact**			
Yes	26 (100.0%)	0 (0%)	–
No	2 (8.7%)	21 (91.3%)	6.12 (3.03–9.21)

^a^Row percentages. ^b^Overall p-values of Wald tests were p-value < 0.01.

**Figure 1:**
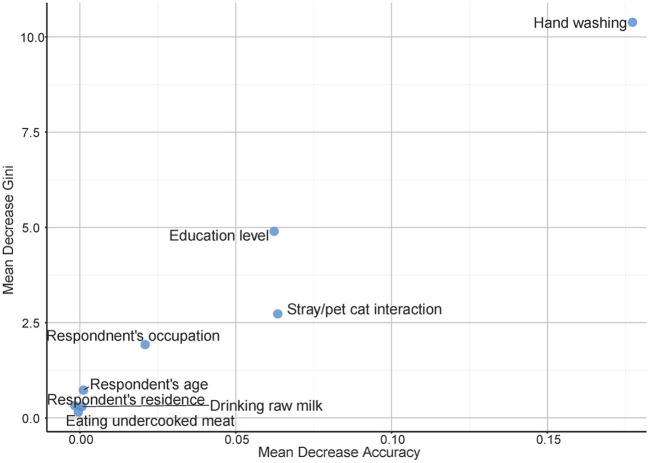
Multi-way variable importance plot from the best random forest model for risk factors associated with toxoplasmosis among pregnant women in Sharqia Governorate, Egypt.

The most crucial covariate identified by the RF model was hand washing after contact with cats which was highly ranked both on effect on model accuracy and Gini index. Other highly ranked factors were the levels of education of the women interviewed and their interactions with pets and stray cats, which also had high odds ratios in Firth logistic regression. Interestingly, neither eating undercooked meat nor drinking raw milk came up as highly important in our study population using this analytical approach (Figure 1).

**Figure 2:**
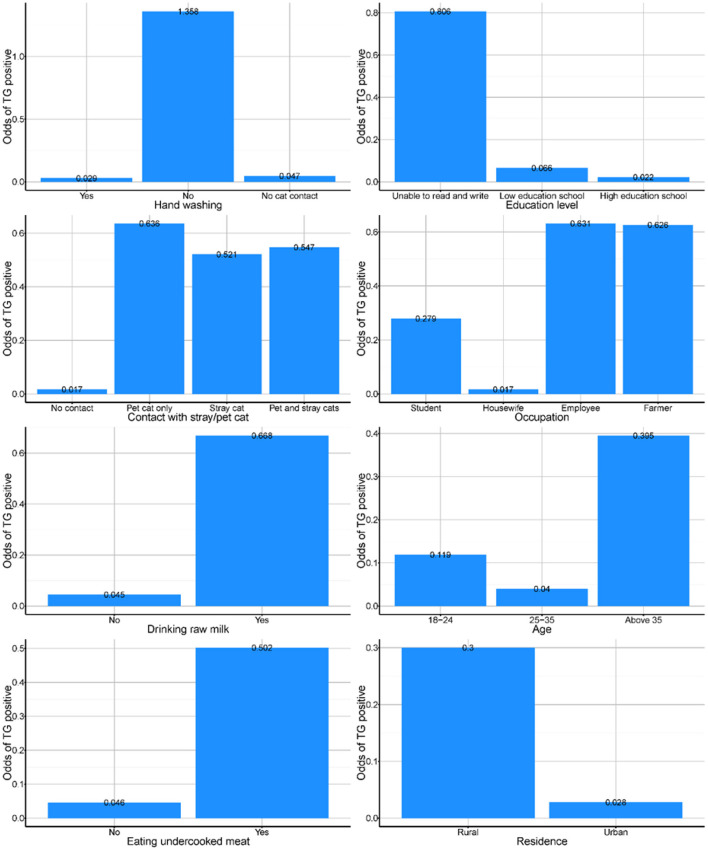
Partial dependency plots from the best random forest model presenting odds of toxoplasmosis among pregnant women exposed to different factors in Sharqia Governorate, Egypt.

From the partial dependency plots, it was evident that failing to wash hands after contact with cats was the riskiest behavior, with the odds of positive *T. gondii* cases in those individuals being 1.3 times high compared to almost negligible odds for women who either washed their hands or had no contact with cats (Figure 2). Additionally, the least educated group of our sample population were more likely to be *T. gondii* positive than any other group. Any contact with cats increased the likelihood of being *T. gondii* positive irrespective of whether the casts were domestic or stray (Figure 2). Similarly, consumption of raw milk and undercooked meat increased the likelihood of testing positive and women residing in rural parts of the Sharqia governorate were also more likely to be *T. gondii* positive than those who lived in urban Sharqia (Figure 2).

Only 29 participants agreed to enroll their domestic cats in the study, of which 5 (17%) were *T. gondii* positive. Additionally, women letting their pet cats outdoors or allowing them to contact other animals, such as dogs or other stray cats or small ruminants, particularly in rural areas, increased the odds of their cats getting infected with *T. gondii*. The use of dry food and litter trays, among other factors, was associated with lower odds of *T. gondii* infection (Table 3).

**Table 3:** A summary of risk factors of pet cats (*n* = 29), univariable firth logistic regression model.

	**–Ve *N* (%)^a^**	**+Ve *N* (%)**	**OR (95%CI)**
**Type of cat food**			
Mixed	12 (75.0)	4 (25.0)	–
Dry	12 (92.3)	1 (7.7)	0.33 (0.04–2.48)
**Use of litter trays**			
Yes	13 (72.2)	5 (27.8)	–
No	11 (100.0)	0 (0.0)	0.11 (0.01–2.14)
**Allow domestic cats outdoors**			
Yes	14 (77.8)	4 (22.2)	–
No	10 (90.9)	1 (9.1)	0.46 (0.06–3.44)
**Contact with other animals**			
Yes	13 (81.3)	3 (18.8)	–
No	11 (84.6)	2 (15.4)	0.84 (0.14–5.09)

^*a*^Row percentages.

## 4. Discussion

Toxoplasmosis, an obligatory intracellular protozoan, is a major global zoonotic disease caused by the protozoan *T. gondii* (phylum Apicomplexa). Deleterious health effects with severe consequences have been associated with toxoplasmosis in immunocompromised individuals and pregnant women (35). It causes chronic illness and clinical symptoms in neonate cats, geriatric, and immunocompromised animals (36).

Concerning risk factors significantly associated with *T. gondii* infection include education, where the odds of being *T. gondii* positive decreased by 83% and 98% for low- and high-educated women, respectively, compared to illiterate women. This finding was similar to Bittencourt et al. (37) and Demiroglu et al. (38) in Brazil Agmas et al. (39), in Ethiopia Alsammani (40), and Olariu et al. (41) who reported that illiterate pregnant women have a higher *T. gondii* infection in Western Romania. In Egypt, illiterate women cannot read and adopt less hygienic practices and are more likely to live in rural areas where they are more likely to be in close contact with animals as compared to educated ones. However, there were findings that there was no relation between the education level of pregnant women and *T. gondii* infection in other African countries (40, 42, 43). Regarding occupation, employees and farmers showed higher odds of *T. gondii* infections compared to students. This result is in line with Mwambe et al. (43), who found that the rate of *T. gondii* infection was higher among employed pregnant women. This association could, however be influenced by the occupation the women were involved in and the associated occupational hazards/risks.

According to hygienic practices related to pregnant women, the proportion of *T. gondii* infection in women who ate raw meat was higher than in those ate cooked ones. This finding agreed with Abdelbaset et al. (14), Ibrahim et al. (21), Nassef et al. (23), Demiroglu et al. (38), van Enter et al. (44), Abamecha and Awel (45), and Eroglu and Asgin (46) in Turkey. Drinking raw milk was more likely to experience *T. gondii* infection than drinking pasteurized or processed milk. This result agreed with Cook et al. (47) while it contrasts with that mentioned by Bahia-Oliveira et al. (48), in Brazil Olariu et al. (41), in Western Romania and Eroglu and Asgin (46) in Turkey.

Considering the contact with cats which are the final host and responsible for spreading of oocysts through feces; our study showed that pregnant women who got in contact with stray and pet cats were associated with higher odds of *T. gondii* infection. This data concurs with results reported by Agmas et al. (39); Abamecha and Awel (45); Negero et al. (49) in Eithiopia, Abdelbaset et al. (14), Ibrahim et al. (21); in Egypt and Olariu et al. (41) in western Romania. However, studies conducted in Turkey and Sri Lanka showed no relationship between toxoplasmosis and cat ownership (50, 51). The difference between the results may be due to various factors, including the difference in the prevalence of toxoplasmosis in cats living in diverse geographic regions, the difference in contact time, and compliance with hygiene requirements. Washing the hand after contact with cats; our study revealed that women who did not wash their hands after contact with cats were more likely to experience *T. gondii* infection than those who washed their hands as recorded by Kapperud et al. (29) in Norway. Given the fecal oral transmission route, hand washing reduced the risks of infection to the women. The high prevalence of *T. gondii* in pregnant women in this study could be the residence of most participants in rural areas, contact with cats and did not follow hygienic measures.

The oocysts of *T. gondii* are excreted for a short period of about 1–3 weeks from infected cats, mostly once in their lifetime (52) this explains the difficulty in diagnosis during single fecal examination. Applying the PCR technique for the detection of *T. gondii* DNA in cat feces is appropriate for a high detection sensitivity, specificity and reproducibility. Also, a sensitive PCR test may be used to detect low numbers of shedding oocysts, which are usually undetectable using traditional microscopy (7).

Regarding risk factors contributed to toxoplasmosis in cat, the use of dry food and litter trays were found to be associated with lower odds of *T. gondii* infection, while the increase of getting an infection due to allowing pet cats outdoors or contact with other animals such as dogs or donkeys with *T. gondii* (53–55). The high prevalence of *T. gondii* in domestic cats in this study is attributed to allow domestic cats outdoors and contact with other animals.

Educating pregnant women about the source of *Toxoplasma* infection, hand hygiene, proper cooking of meat products and hygienic measure for cleaning the cat litter box, keeping cats indoors away from animals and stimulating cats to use the litter box are important as preventive and control strategy.

## 5. Conclusion

As far as we know, this is the first study to use the molecular detection of *T. gondii* in both humans and cats in the Sharqia Governorate, Egypt. Education, occupation, drinking raw milk, contact with stray and pet cats and eating under cooked meat were significantly associated with the presence of *T. gondii* infections. Washing hands constitutes a serious yet preventable public health concern that requires targeted, community-specific efforts. Cat owners, particularly pregnant women, need to be aware about the risk of *T. gondii* infection while handling cats and pets owner should be advised to take necessary hygienic measure to prevent the infection.

## Data availability statement

The original contributions presented in the study are included in the article/Supplementary material, further inquiries can be directed to the corresponding author.

## Ethics statement

The studies involving human participants were reviewed and approved by Committee of the Faculty of Veterinary Medicine, Zagazig University, Egypt (ethical approval number: ZU-IACUC/2/F/205/2022). The patients/participants provided their written informed consent to participate in this study. The animal study was reviewed and approved by Committee of the Faculty of Veterinary Medicine, Zagazig University, Egypt (ethical approval number: ZU-IACUC/2/F/205/2022). Written informed consent was obtained from the owners for the participation of their animals in this study. Written informed consent was obtained from the individual(s) for the publication of any potentially identifiable images or data included in this article.

## Author contributions

All authors donated to the study's design, methodology, data collection statistical analysis, and manuscript editing and writing. All authors contributed to the article and approved the submitted version.
