# Supplementary material for: Interplay between cross sectional analysis of risk factors associated with Toxoplasma gondii infection in pregnant women and their domestic cats
Source: Front Vet Sci. 2023 Mar 24;10:1147614. doi: 10.3389/fvets.2023.1147614 (PMC10080162; doi:10.3389/fvets.2023.1147614)
Supplement: Supplementary file 1 [file Data_Sheet_1.docx]

**Table S1**. An English-translated version of the questionnaires.

|  | Characteristics |
| --- | --- |
| **Socio-demographic data** |  |
| Age |  |
| Residence |  |
| Education |  |
| Occupation |  |
| Pregnancy stage |  |
| Previous abortion history |  |
|  |  |
| Can individuals acquire toxoplasmosis by touching sand/soil in the garden or yard? | yes |
|  | No |
| Do pregnant women develop serious complications from toxoplasmosis? | yes |
|  | No |
| Can toxoplasmosis in pregnant women cause no symptoms? | yes |
|  | No |
| Do you routinely wash your hands after gardening? | yes |
|  | No |
| Do you routinely wash your hands after changing the cat litter box? | Yes |
|  | No |
| Do you routinely wash your hands after handling raw meat? | Yes |
|  | No |
| Do you thoroughly cook meat before consumption? | Yes |
|  | No |
| Do you have direct contact with a cat? | Yes |
|  | No |
| Do you avoid stray cats? | Yes |
|  | No |
| Do you wear personal protective equipment while handling your cat? | Yes |
|  | No |
| Do you feed your cat dry or commercial food and not let it kill and eat rodents? | Yes |
|  | No |

| Target gene | Primers sequences | Amplified segment (bp) | Primary  denaturation | Amplification (40 cycles) | | | Dissociation curve  (1 cycle) | | |
| --- | --- | --- | --- | --- | --- | --- | --- | --- | --- |
|  |  |  |  | Secondary denaturation | Annulation | Extention | Secondary denaturation | Annulation | Final denaturation |
| B1 fragment | 3-׳CGCTGCAGGGAGGAAGACGAAAGTTG-5׳ | 529 | 94˚C  10 min. | 94˚C  45 sec. | 55˚C  45 sec. | 72˚C  45sec. | 94˚C  1 min. | 55˚C  1 min. | 94˚C  1 min. |
|  | 5-׳CGCTGCAGACACAGTGCATCTGGATT-3׳ |  |  |  |  |  |  |  |  |

**Table S 2**: Primers sequences, target genes, amplicon sizes and cycling conditions (Tavassoli et al., 2013).
